# Supplementary material for: Nano formulation development and antibacterial activity of cinnamon bark extract-chitosan composites against Burkholderia Glumae the causative agent of Bacterial Panicle Blight in rice
Source: PLoS One. 2025 Jun 20;20(6):e0320032. doi: 10.1371/journal.pone.0320032 (PMC12180725; doi:10.1371/journal.pone.0320032)
Supplement: S1 File — (DOCX) [file pone.0320032.s001.docx]

Supporting Information


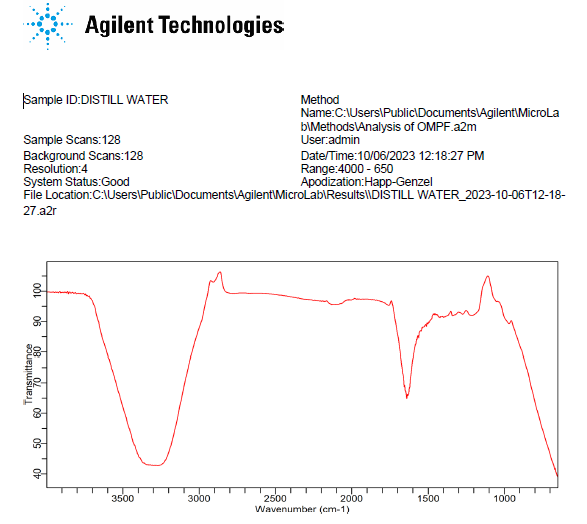


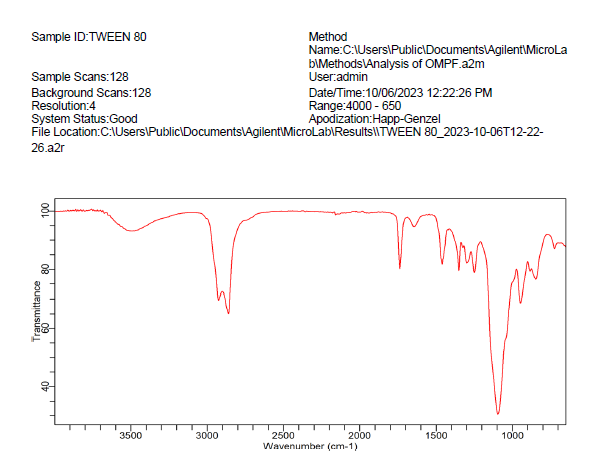


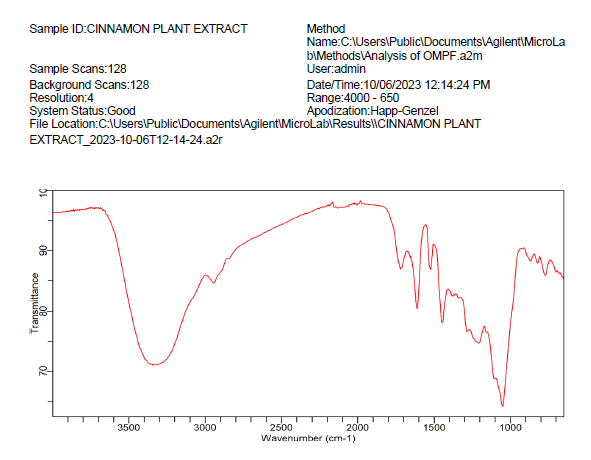


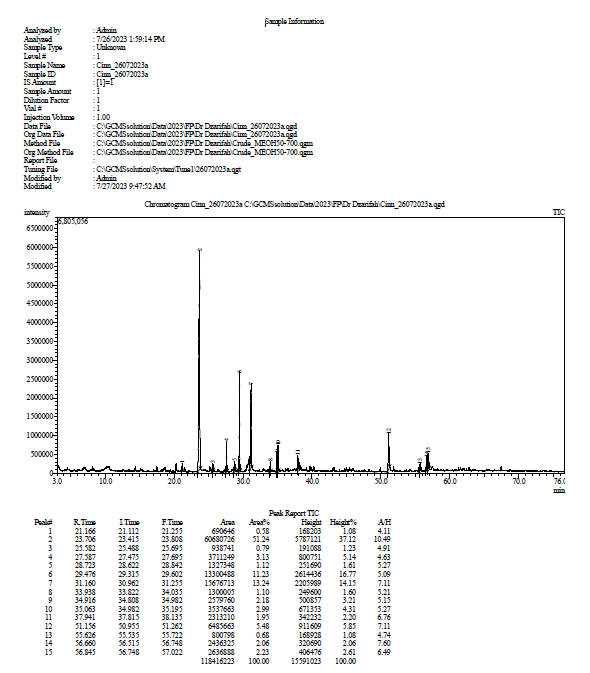


Plant extract zone of inhibition data

| The SAS System |
| --- |

| **Obs** | **rep** | **trt** | **y** |
| --- | --- | --- | --- |
| **1** | 1 | 1 | 17 |
| **2** | 1 | 2 | 15 |
| **3** | 1 | 3 | 12 |
| **4** | 1 | 4 | 10 |
| **5** | 1 | 5 | 20 |
| **6** | 1 | 6 | 0 |
| **7** | 2 | 1 | 18 |
| **8** | 2 | 2 | 16 |
| **9** | 2 | 3 | 13 |
| **10** | 2 | 4 | 11 |
| **11** | 2 | 5 | 20 |
| **12** | 2 | 6 | 0 |
| **13** | 3 | 1 | 17 |
| **14** | 3 | 2 | 16 |
| **15** | 3 | 3 | 12 |
| **16** | 3 | 4 | 11 |
| **17** | 3 | 5 | 20 |
| **18** | 3 | 6 | 0 |

| The SAS System |
| --- |

The ANOVA Procedure

| **Class Level Information** | | |
| --- | --- | --- |
| **Class** | **Levels** | **Values** |
| **trt** | 6 | 1 2 3 4 5 6 |

| **Number of Observations Read** | 18 |
| --- | --- |
| **Number of Observations Used** | 18 |
