# Supplementary material for: Nano formulation development and antibacterial activity of cinnamon bark extract-chitosan composites against Burkholderia Glumae the causative agent of Bacterial Panicle Blight in rice
Source: PLoS One. 2025 Jun 20;20(6):e0320032. doi: 10.1371/journal.pone.0320032 (PMC12180725; doi:10.1371/journal.pone.0320032)
Supplement: Attachment — Submitted filename: REBUTTAL LETTER.docx [file pone.0320032.s004.docx]

**REBUTTAL LETTER**

**Dear Editor-in-Chief of PLOS ONE,**

I am writing to inform you that I have not received any comments from the editors regarding my manuscript **PONE-D-24-55598**. However, I have decided to withdraw it in order to improve the manuscript, incorporate additional results related to the greenhouse, and enhance its accuracy and comprehensiveness.

Once the necessary amendments are made and the research is completed in the best possible way, I sincerely hope that you will consider my revised manuscript for publication in PLOS ONE.

Thank you for your time and consideration. I appreciate your support and look forward to your response.

**With my sincere respect,**
QAMAR MOHAMMED NAJI
gs64360@student.upm.edu.my
