# Supplementary material for: Nano formulation development and antibacterial activity of cinnamon bark extract-chitosan composites against Burkholderia Glumae the causative agent of Bacterial Panicle Blight in rice
Source: PLoS One. 2025 Jun 20;20(6):e0320032. doi: 10.1371/journal.pone.0320032 (PMC12180725; doi:10.1371/journal.pone.0320032)
Supplement: Attachment — Submitted filename: Plos one review.docx [file pone.0320032.s002.docx]

**Corresponding Author: Qamar Mohammed Alsutan, phD :"phD" → Use "PhD"**

**Line 26: "CBE-CS" →** Ensure consistent hyphenation (sometimes written as "CBE- CS" with space).

**Line 38&49:** "Burkholderia. glumae" → Remove erroneous period: "Burkholderia glumae."

**Line 104**: "chitosan, and various chemicals were sourced from local markets" → Include catalog number and supplier location.

**Line 117**: "exposed to 60°C in a water bath for 30 minutes to evaporate the methanol" → Confirm complete solvent removal (residual methanol may affect bioassays).

**Line 142:** “evaluate” → “evaluation”.

**Line 175:** The section is misnumbered as “1.1.4” and should be consistent with "2.1.4".

**Line 198:** Reference to "Maluin et al. (2019)" – Confirm if this is an in-press or unpublished work.

**Line 251**: "*B. glumae communis*" → Clarify strain name (Should it be *B. glumae*?).

**Line 316:** "P<0.05P" → extra "P" at end.

**Line 328:** Mean n=3" is out of place. Should be embedded in the caption or footnote.

**Line 330-332:** "minimum inhibitory concentration (MIC) of 6.25 µg/mL" vs. **Line 330**: "MIC of 6.25 mg/mL" → Units conflict (µg vs. mg). Resolve inconsistency.

**Line 480:** “Determine of antibacterial activity” → revise to “Determination of antibacterial activity”.

**Line 571-575**: Overemphasis on volatile compounds without linking to nanoformulation stability. Discuss how encapsulation preserves these volatiles.

**Table 5**: "Disease severity" values lack units (e.g., % or scale).

**Figure Legends:** All figures lack scale bars (e.g., Fig. 6: "10,000×" magnification is insufficient without a scale bar).

**Were any phytotoxicity or cytotoxicity evaluations conducted beyond plant growth traits?**
