# Supplementary material for: Nano formulation development and antibacterial activity of cinnamon bark extract-chitosan composites against Burkholderia Glumae the causative agent of Bacterial Panicle Blight in rice
Source: PLoS One. 2025 Jun 20;20(6):e0320032. doi: 10.1371/journal.pone.0320032 (PMC12180725; doi:10.1371/journal.pone.0320032)
Supplement: Attachment — Submitted filename: PONE-D-25-07190.docx [file pone.0320032.s003.docx]

**Dear Editor in chief of PlosOne**

Thank you for considering me as reviewer for your valuable journal. Regarding the submission PONE-D-25-07190 “Nano formulation development and antibacterial activity of cinnamon bark extract-chitosan composites against *burkholderia glumae*, the causative agent of bacterial panicle blight in rice”

However, the study design and techniques sound of interest, the writing style needs fundamental and serious improvements. My comments are as follows

Bests

Zahra Hesari

- Keywords: add chitosan to keywords
- Section 2. Material and method: (lines 104-109) is written poorly, containing un informative sentences. Chitosan and all chemicals are bought from local market with no specific brand and manufacturing company? This may lead to a non-reliable and non-repeatable results.
- Needs English language corrections. e.g. line 117 filtered s correct instead of filters.
- What is “inhibitory zone of inhibition”? (L 121) and (L123)
- “Bacteria growth evaluate” authors mean Bacterial growth evaluation???? (L142)
- In section II. Assessment of MIC and MBC, how much microorganism suspension was added?
- Sentences like “The resulting spectra and peaks were observed on a computer screen” are not necessary and can be deleted. (L 173)
- Section 2.2.1, results are presented in method section.
- Section 2.2.2, duration of stability studies is not mentioned.
- What was the λmax for EE and LC calculation? Not a range (200-400)
- Just one disk diffusion or MIC and MBC determination section was enough for both extract and nano formulation. These sections are repeated in method section for extract and nano formulation separately.
- Statistical analysis should be mentioned in method part, not in result section.
- FTIR spectra should be provided for both CBE and CBE chitosan nano-formulation.
- Figures need more quality and resolutions. Fig 2 is not informative and can be omitted.
- The writing in section 2 and 3 should be completely restructured.
- Discussion is poorly written with no discussing about the results of other studies.
- Conclusion: There is no need to explain about method in conclusion. Add the limitations.
