# Supplementary material for: Nano formulation development and antibacterial activity of cinnamon bark extract-chitosan composites against Burkholderia Glumae the causative agent of Bacterial Panicle Blight in rice
Source: PLoS One. 2025 Jun 20;20(6):e0320032. doi: 10.1371/journal.pone.0320032 (PMC12180725; doi:10.1371/journal.pone.0320032)
Supplement: Attachment — Submitted filename: Response to Reviewers.docx [file pone.0320032.s005.docx]

**Response to Reviewers**

**Dear Editor,**

We would like to express our sincere gratitude for the valuable feedback provided by the reviewers on our manuscript entitled: **“Nanoformulation development and antibacterial activity of cinnamon bark extract-chitosan composites against *Burkholderia glumae*, the causative agent of bacterial panicle blight in rice”** (Manuscript ID: **PONE-D-25-07190**).

We have carefully considered all the comments and suggestions provided by the reviewers and have revised the manuscript accordingly. Detailed responses to each comment have been provided in the revised version, and all necessary modifications have been made to improve the clarity, methodology, and overall quality of the manuscript.

We believe that the revised version now addresses all the concerns raised and hope that it meets the publication standards of PLOS ONE.

Thank you again for the opportunity to revise our work. We look forward to your positive response.

|  | **Journal Requirements:** | |  |
| --- | --- | --- | --- |
| **NO** | **Reviewer Comments** | **Author Procedures** | **Line** |
| 1 | Please ensure that your manuscript meets PLOS ONE's style requirements, including those for file naming. | In response to the reviewer’s comment, we have ensured that the manuscript has been thoroughly updated to meet the PLOS ONE style requirements.Top of Form  Bottom of Form | All manus. |
| 2 | We note that you have provided funding information that is not currently declared in your Funding Statement. However, funding information should not appear in the Acknowledgments section or other areas of your manuscript. | We have edited the Acknowledgment section to exclude financial disclosures, ensuring alignment with PLOS ONE’s requirements. | 644-646 |
| 3 | Please confirm at this time whether or not your submission contains all raw data required to replicate the results of your study. Authors must share the “minimal data set” for their submission. | All raw data required to reproduce the findings of this study have been included in the submission. | All |
| 4 | PLOS requires an ORCID iD for the corresponding author in Editorial Manager on papers submitted after December6th, 2016. | The ORCID iD of the corresponding author has been provided. | Line 16 |
| 5 | Please upload a new copy of Figure 7 as the detail is not clear. | A new copy of Figure 7 has been uploaded with clear detail. | Fig 7 |
|  | **Review Comments** | | |
|  | **Reviewer 1** |  |  |
| 1 | The manuscript is poorly written with several grammatical and typographical errors. The flow of ideas is difficult to follow, and the writing lacks cohesion. | Many grammatical and typographical errors in the manuscript have been corrected as much as possible, and an attempt has been made to reconstruct the sequence of ideas and increase the coherence of the text. | All lines |
| 2 | Title Formatting: The title contains italics, which are unnecessary. Italics should only be used for bacterial names, and even in this case, the first letter of the genus should be capitalized, and no period is needed when writing the full genus name. | The title has been reformatted to use italics only for bacterial names, capitalize the first letter of the genus name, and remove the full period from the genus name. | 2-4 |
| 3 | Figure Legends need to be written in a standard format. The current legends are not informative and lack essential details. For example, Figure 2 does not specify what the other wells represent after 3.125, and there are no labels for 2B.Additionally, Figure 1 lacks any kind of legend or explanation. Clear and concise figure legends are critical to help the reader interpret the figures properly. | The figure descriptions have been rewritten to address valuable reviewer comments.  The title of Figure 2 has been revised to reflect what other wells represent. In addition,  Figure 1 has been reinterpreted. | 336-339  216-220 |
| 4 | Inconsistent Sections: In Line 312, the manuscript moves into the "Results and Discussion" section, but Line 313continues discussing statistical analysis without the appropriate title. This is confusing and suggests lack of attention to important details while writing the manuscript. | The paragraph after the results and discussion section was reformulated to show the type of procedures followed and the statistical programs used to obtain the results and moved to methodology section. | 113-117 |
| 5 | The aspect ratio of the figures in Figure 7 should be consistent. The figures appear stretched or distorted, which impacts their clarity. The authors should ensure that all figures are presented with a consistent aspect ratio and that they are visually clear. | Due to the large volume of results obtained, and to present the most relevant findings, multiple microscopic images were combined in Figure 7. This combination slightly affected the image clarity and accuracy. Nevertheless, the images effectively illustrated the key morphological changes in cell shape. Efforts were made to adjust the image dimensions and incorporate higher-quality, more accurate images in the final version of Figure 7 | Fig 7 |
| 6 | Scientific Errors: There appear to be several scientific inaccuracies within the manuscript. For ex: It is unclear why the positive control used in Figure 2 was also not used in the greenhouse studies. Using streptomycin in plant studies would strengthen the data. | In this research and the associated greenhouse experiments, six study models were utilized, as outlined in Section 3.2.1. Three of the models were treated with varying concentrations of the developed nano-extract. The GHSBS model served as the positive control, while the GHSB model represented the negative control. Data from all six models were included to provide a comprehensive analysis and strengthen the overall findings | 264-269 |
|  | **Reviewer 2** | | |
| 1 | chitosan composites against Burkholderia glumae, the causative agent of bacterial panicle blight in rice"presents an interesting and relevant approach to developing natural antimicrobial formulations for agricultural pathogens.The use of cinnamon bark extract combined with chitosan for nanoformulation and its evaluation against B. glumae holdspromise for sustainable disease management in rice | Thank you for your positive and encouraging feedback. We appreciate your recognition of the relevance and potential impact of our work. The combination of cinnamon bark extract with chitosan in a nanoformulated composite was indeed selected to explore an environmentally friendly and sustainable strategy for managing *Burkholderia glumae*, a significant pathogen in rice cultivation. We are encouraged that the reviewer finds this approach promising for agricultural disease control, and we hope our findings contribute meaningfully to the development of natural alternatives in plant protection. | All |
|  | **Reviewer 3** |  |  |
| 1 | Keywords: add chitosan to keywords | Chitosan has been added to keywords | 44 |
| 2 | Section 2. Material and method: (lines 104-109) is written poorly, containing uninformative sentences. Are Chitosan and all chemicals bought from local market with no specific brand and manufacturing company? This may lead to non-reliable and non-repeatable results. | We acknowledge the concern regarding the clarity and specificity of the information provided in this section. To address this, we have revised the relevant sentences to include the source, brand, and manufacturer details of the materials used. This will enhance the reproducibility and reliability of the study. | 105-112 |
| 3 | Needs English language corrections. e.g. line 117 filtered s correct instead of filters. | The world filters has been corrected to filtered. | 123 |
| 4 | What is “inhibitory zone of inhibition”? (L 121) and (L123) | Thank you for your valuable feedback. It has been modified to the "zone of inhibition” | 128 & 130 |
| 5 | “Bacteria growth evaluate” authors mean Bacterial growth evaluation???? (L142) | Thank you for your valuable feedback. It has been removed. The title became " Bacterial growth” | 152 |
| 6 | In section II. Assessment of MIC and MBC, how much microorganism suspension was added? | The paragraph was modified to read” A suspension of *B. glumae* adjusted to an optical density of 0.15 at 600 nm (~1 × 10⁶ CFU/mL) was added to each well (10 μL)” | 144 |
| 7 | Sentences like “The resulting spectra and peaks were observed on a computer screen” are not necessary and can be deleted. (L 173) | The sentence “The resulting spectra and peaks were observed on a computer screen” has been deleted. | 186 |
| 8 | Section 2.2.1, results are presented in method section | The sentence “forming chitosan nanoparticles (200–1000 nm, zeta potential 20–60 mV)” has been deleted from section 2.2. | 124 |
| 9 | Section 2.2.2, duration of stability studies is not mentioned. | In our study, the duration of stability studies in particle size analysis was 72 hours, and this was added to Section I- Particle size analysis. | 235 |
| 10 | What was the λmax for EE and LC calculation? Not a range (200-400) | λ_max_ is the maximum absorbance wavelength of bioactive compound (e.g., cinnamaldehyde in CBE). It is determined by scanning the extract in a UV-Vis spectrophotometer, and its range typically between 200–600 nm.  In CBE it is between ~290–300 nm, Cinnamaldehyde (major component) ~290–295, Phenolic compounds ~270–320. | 243-244 |
| 11 | Just one disk diffusion or MIC and MBC determination section was enough for both extract and nano formulation. These sections are repeated in the method section for extract and nano formulation separately. | Valuable note, section 2.2.4 has been deleted.  The equation for calculating the antibacterial effect using the inhibition formula (1) has been added to Section 2.1.2. | 137-138 |
| 12 | Statistical analysis should be mentioned in the method part, not in the result section. | In response to the comments from Reviewers 1 and 3, the Statistical Analysis section has been revised to provide a clearer and more detailed explanation of the data analysis methods and calculation procedures used, ensuring alignment with the reviewers' suggestions and enhancing the overall clarity of the methodology | 113-117 |
| 13 | FTIR spectra should be provided for both CBE and CBE chitosan nano-formulation | The FTIR spectral results for both CBE and CBE-CS were obtained; however, due to data redundancy and the extensive volume of results, which could impact on the overall length and focus of the manuscript, only the FTIR analysis of CBE is presented in this paper. The FTIR data for CBE-CS is available from the authors upon request. | All |
| 14 | Figures need more quality and resolutions. Fig 2 is not informative and can be omitted | The accuracy and clarity of the figures have been adjusted as much as possible, and Figure 2 has been adjusted. | All figs. |
| 15 | The writing in section 2 and 3 should be completely restructured | Sections 2 and 3 have been completely restructured. | Sec. 2 & 3 |
| 16 | Discussion is poorly written with no discussion about the results of other studies | The discussion section has been rewritten, considering the results of other studies. | 578-614 |
| 17 | Conclusion: There is no need to explain about method in conclusion. Add the limitations. | The conclusion section has been rewritten, considering the limitation. | 616-640 |
